# Supplementary material for: Assessing the feasibility of sustaining SARS-CoV-2 local containment in China in the era of highly transmissible variants
Source: BMC Med. 2022 Nov 15;20:442. doi: 10.1186/s12916-022-02640-6 (PMC9666984; doi:10.1186/s12916-022-02640-6)
Supplement: Supplementary file 1 — Additional file 1: Figure S1. Epidemic trajectory, vaccine uptake, and clinical severity of Hong Kong SAR’s Omicron wave. Figure S2. Vaccinations uptake by age in Hong Kong SAR, China, mainland China and New Zealand. Figure S3. Population immunity levels in the baseline immunization scenario and the enhanced immunization scenarios. Figure S4. The observed epidemic growth rate and effective reproduction numbers with baseline interventions in the early stage of the two recent outbreaks in mainland China. Figure S5. Relative disease burden of SARS-CoV-2 under different counterfactual mitigation scenarios in mainland China (sensitivity analysis). Table S1. Summary of epidemiological parameters for the SARS-CoV-2 Omicron variant. Table S2. Effectiveness of COVID-19 vaccines. Table S3. The hypothetical origin-destination mobility matrix of NPI intensity Level 0-4. Table S4. The hypothetical origin-destination mobility matrix of NPI intensity Level 5. Table S5. List of considered hypothetical mitigation scenarios. [file 12916_2022_2640_MOESM1_ESM.docx]

Additional File 1

for

**Assessing the feasibility of sustaining SARS-CoV-2 local containment in China in the era of highly transmissible variants**

Yan Wang^1#^, Kaiyuan Sun^2#^, Zhaomin Feng^3^, Lan Yi^1^, Yanpeng Wu^1^, Hengcong Liu^1^, Quanyi Wang^3^, Marco Ajelli^4^, Cécile Viboud^2^, Hongjie Yu^1*^

**Author Affiliations:**

1. School of Public Health, Fudan University, Key Laboratory of Public Health Safety, Ministry of Education, Shanghai, China.
2. Division of International Epidemiology and Population Studies, Fogarty International Center, National Institutes of Health, Bethesda, MD, USA.
3. Beijing Center for Disease Prevention and Control (CDC), Beijing, China.
4. Laboratory of Computational Epidemiology and Public Health, Department of Epidemiology and Biostatistics, Indiana University School of Public Health, Bloomington, IN, USA.

^#^ These authors contributed equally.

^*^ Corresponding Author: [yhj@fudan.edu.cn](mailto:yhj@fudan.edu.cn)


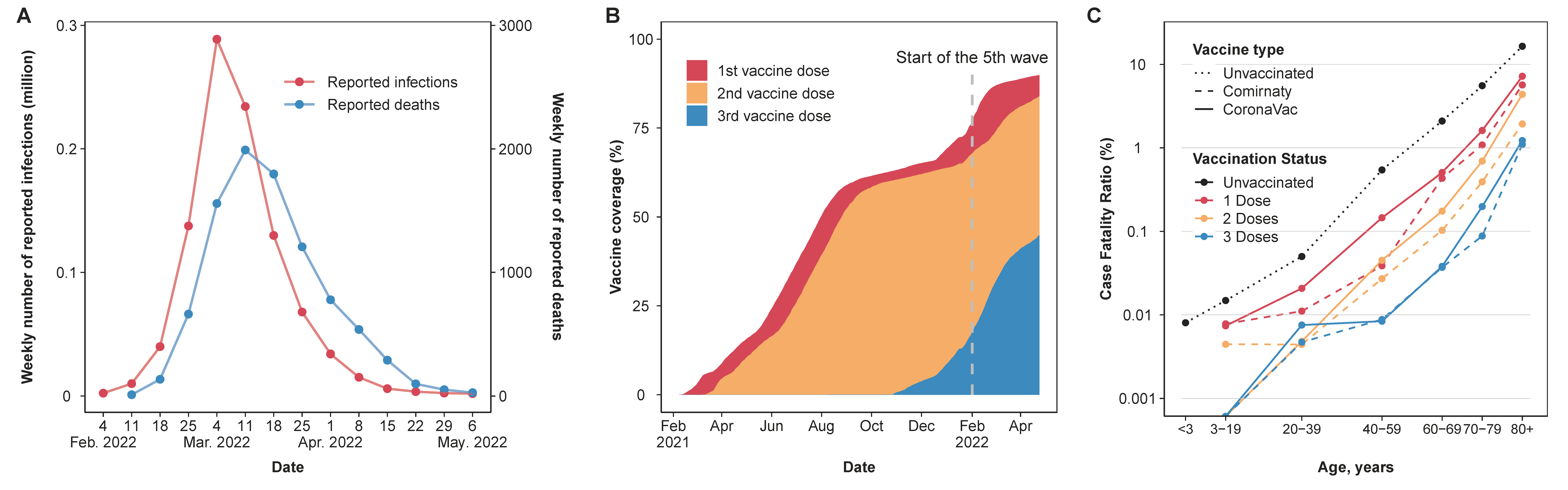


## Fig. S1. Epidemic trajectory, vaccine uptake, and clinical severity of Hong Kong SAR’s Omicron wave. A, Weekly incidence of reported Omicron infections and deaths from February 1 to May 9, 2022. B, Vaccine coverage in Hong Kong until May 8, 2022 stratified by vaccine dose. C, Hong Kong’s crude case fatality ratio by age and vaccination status.





## Fig. S2. Vaccinations uptake by age in Hong Kong SAR, China (as of February 15, 2022), mainland China (as of March 17, 2022) and New Zealand (as of September 20, 2022). A, Primary vaccination. B, Booster vaccination.





## Fig. S3. Population immunity levels in the baseline immunization scenario and the enhanced immunization scenarios. We assume the baseline immunization scenario with the same primary vaccine coverage as that of New Zealand (as of September 20, 2022) and 0% booster coverage. We assume the two enhanced immunization scenarios (one with homologous booster and the other with heterologous booster) with the same primary and booster vaccine coverage as that of New Zealand as of September 20, 2022. However, we made two adjustments for the younger age groups to be more aligned with mainland China’s current COVID-19 immunization policy. Specifically, for population aged 4-11 years, we assume they have the same primary vaccine coverage as those aged 12-17 years in New Zealand, as mainland China has approved COVID-19 vaccination for this age group. We assume a booster vaccine coverage of 0% for those aged 0-17 years, as booster for this age range are currently not part of the national COVID-19 vaccination programme in China.





## Fig. S4. The observed epidemic growth rate and effective reproduction numbers with baseline interventions in the early stage of the two recent outbreaks in mainland China. A, the outbreak in Pudong district, Shanghai. Red dots are daily new infections and the blue line best fitted growth rate. The blue shade represents 95%CI. B, Same as a but for the outbreak in Jilin city, Jilin province.





## Fig. S5: Relative disease burden of SARS-CoV-2 under different counterfactual mitigation scenarios in mainland China. As a sensitivity analysis, we provide relative disease burden of each scenario based on the upper bound projections. The results based on the lower bound projections are shown in Fig. 4.

## Table S1. Summary of epidemiological parameters for the SARS-CoV-2 Omicron variant

| **Parameter** | **Value** |
| --- | --- |
| Basic reproduction number ($R_{0}$) | Omicron BA.1: 7.5  Omicron BA.2: 9.5 |
| Dispersion parameter ($k$) | 0.43 |
| Incubation period ($\tau_{incu}$) | mean±sd: 5.8±3.0 days |
| Generation interval ($\tau_{GI}$) | mean±sd:4.7±1.3 days |
| Age-specific asymptomatic rate ($\Phi_{asymp}$) | 0-19 years: 81.9%  20-39 years: 77.6%  40-59 years: 69.5%  60-79 years: 64.5%  80+ years: 35.4% |

## Table S2. Effectiveness of COVID-19 vaccines.

| Vaccine effectiveness (*VE*) | Primary vaccination | Homologous booster | Heterologous booster |
| --- | --- | --- | --- |
| Against onward transmission (${VE}_{T}$) | 0% | 10.6% | 10.6% |
| Against infection (${VE}_{I}$) | 14.8% | 18.9% | 52.7% |
| Against symptomatic disease (${VE}_{S}$) | 21.5% | 24.3% | 57.0% |

## Table S3. The hypothetical origin-destination mobility matrix of NPI intensity Level 0-4.

| Risk level of the origin street/town | Risk level of the destination street/town | | | Mobility within one street/town |
| --- | --- | --- | --- | --- |
|  | High | Moderate | Low |  |
| High | 0 | 0.1 | 0.3 | 0.3 |
| Moderate | 0.1 | 0.3 | 0.5 | 0.6 |
| Low | 0.3 | 0.5 | 0.8 | 0.9 |

Note: The risk level refers to the real-time risk level of the street/town at the time of the transmission event occurring. The value in each cell of the matrix refers to the average travel probability per person after official report of the first detected infection, given the risk level of the origin and destination region.

## Table S4. The hypothetical origin-destination mobility matrix of NPI intensity Level 5.

| Risk level of the origin street/town | Risk level of the destination street/town | | | Mobility within one street/town |
| --- | --- | --- | --- | --- |
|  | High | Moderate | Low |  |
| High | 0 | 0 | 0 | 0 |
| Moderate | 0 | 0.1 | 0.3 | 0.3 |
| Low | 0.1 | 0.3 | 0.5 | 0.6 |

Note: The risk level refers to the real-time risk level of the street/town at the time of the transmission event occurring. The value in each cell of the matrix refers to the average travel probability per person after official report of the first detected infection, given the risk level of the origin and destination region.

## Table S5. List of considered hypothetical mitigation scenarios.

| **Mitigation scenario** | | **Definition** |
| --- | --- | --- |
| **Variant type** | **Baseline variant**  **(Omicron BA.2)** | The Omicron BA.2 variant with age and vaccine status specific infection fatality ratios (IFRs) estimated based on the Hong Kong Omicron BA.2 wave. |
|  | **Variant with increased disease severity** | A hypothetical more severe variant with age and vaccine status specific IFRs 50% higher than those of the Omicron BA.2. |
|  | **Variant with enhanced immune evasion** | A hypothetical immune evasive variant with age specific IFRs the same as those of Omicron BA.2 for the unvaccinated individuals but 100% higher among vaccinated individuals. |
| **Vaccine coverage** | **Current** | The mainland China’s age-specific vaccination coverage as of March 17^th^, 2022 (Fig. S2). |
|  | **High** | The New Zealand’s age-specific vaccination coverage as of September 20, 2022 (Fig. S2-S3). |
| **Antiviral uptake** | **0%** | Assuming patients at high-risk of SASR-CoV-2 severe outcomes could be treated timely with antiviral of 90% effectiveness against death with 0% uptake. |
|  | **30%** | Assuming patients at high-risk of SASR-CoV-2 severe outcomes could be treated timely with antiviral of 90% effectiveness against death with 30% uptake. |
|  | **70%** | Assuming patients at high-risk of SASR-CoV-2 severe outcomes could be treated timely with antiviral of 90% effectiveness against death with 70% uptake. |
| **Time scale** | **Short term** | Disease burden after one epidemic wave of SASR-CoV-2 with the epidemic size roughly the same as that of the Hong Kong Omicron wave, i.e., 60% overall infection attack rate. For simplicity, we assume infection attack rates remain the same (60%) across all age groups and vaccination status. We do not consider reinfections. |
|  | **Long term** | Long term disease burden of a SARS-CoV-2 variant’s primary infections, assuming 95% of the population have been infected with SARS-CoV-2. For simplicity, we did not account for the disease burden due to repeated infections after the primary ones. |
